# Challenges of Multidrug-resistant New Delhi Metallo-beta-Lactamase (NDM-1)-producing Enterobacteriaceae in Kidney Transplant Patients

**DOI:** 10.1590/2175-8239-JBN-2021-0033

**Published:** 2021-10-15

**Authors:** Renato Demarchi Foresto, Lucas Marengo Menezes, Laura Tomoko Nishimura, Marina Pontello Cristelli, Laila Almeida Viana, Daniel Wagner de Castro Lima Santos, Lúcio R. Requião-Moura, Helio Tedesco-Silva, Jose Medina-Pestana

**Affiliations:** 1 Hospital do Rim, São Paulo, SP, Brasil.; 2 Universidade Federal de São Paulo, Divisão de Nefrologia, São Paulo, SP, Brasil.

**Keywords:** Drug Resistance, Bacterial Infections, Drug Resistance, Bacterial, Kidney Transplantation, Resistência a Medicamentos, Infecções Bacterianas, Farmacorresistência Bacteriana, Transplante de Rim

## Abstract

**Background:**

The emergence of multidrug-resistant NDM-1-producing enterobacteriaceae strains has become a threat to inpatients, especially to immunosuppressed ones, such as kidney transplant recipients. NDM-1 is a carbapenemase that makes gram-negative bacteria resistant to many types of antibiotics. The incidence of carbapenemase-producing enterobacteria infection in solid organ transplant recipients is around 3 to 10%, with a mortality rate of up to 30%.

**Methods:**

We present a case series of 4 patients with NDM-1-producing enterobacteria isolated in urine cultures or rectal swabs. We also conducted a cross-sectional study 30 days after patient identification, collecting surveillance cultures (rectal swab) from all inpatients to assess the extent of spread of this resistance mechanism; a total of 101 patients were included.

**Results:**

Two patients were adequately treated with negative control cultures. The other two patients were not treated because they were asymptomatic and had subsequent negative urine cultures. No new colonization was identified in the cross-sectional screening, and no new cases of urinary NDM-1 infection were recorded after a 4-year follow-up.

**Conclusion:**

Surveillance for infections caused by multidrug-resistant strains in hospitals treating immunosuppressed patients should be continued and prompt action should be taken in cases of outbreaks of multidrug-resistant infections.

## Resumo

**Histórico:**

O surgimento de cepas multirresistentes de enterobacteriaceae produtoras de NDM-1 tornou-se uma ameaça para pacientes hospitalizados, especialmente para os imunossuprimidos, como os receptores de transplante renal. NDM-1 é uma carbapenemase que torna as bactérias gram-negativas resistentes a muitos tipos de antibióticos. A incidência de infecção por enterobactérias produtoras de carbapenemas em receptores de transplante de órgãos sólidos é de cerca de 3 a 10%, com uma taxa de mortalidade de até 30%.

**Métodos:**

Apresentamos uma série de casos de 4 pacientes com enterobactérias produtoras de NDM-1 isoladas em culturas de urina ou esfregaços retais. Também realizamos um estudo transversal 30 dias após a identificação do paciente, coletando culturas de vigilância (esfregaço retal) de todos os pacientes internados para avaliar a extensão de disseminação deste mecanismo de resistência; foram incluídos um total de 101 pacientes.

**Resultados:**

Dois pacientes foram tratados adequadamente com culturas de controle negativo. Os outros dois pacientes não foram tratados porque eram assintomáticos e tiveram culturas de urina negativas subsequentes. Não foi identificada nenhuma nova colonização na triagem transversal, e não foram registrados novos casos de infecção urinária por NDM-1 após um acompanhamento de 4 anos.

**Conclusão:**

A vigilância de infecções causadas por cepas multirresistentes em hospitais que tratam pacientes imunossuprimidos deve ser continuada e devem ser tomadas medidas imediatas em casos de surtos desses tipos de infecções.

## Introduction

The New Delhi Metallo-beta-lactamase (NDM) bacterial enzyme was first identified in 2009 in Sweden in a patient who had previously been hospitalized in New Delhi, India 1. This enzyme is a class B Metallo-beta-lactamase (MBL) produced by bacteria that hydrolyzes almost all clinically available β-lactam antibiotics, including carbapenems, but maintains sensitivity to polymyxins, phosphomycin, aminoglycosides, and tigecycline. The in-hospital dissemination of these bacteria is rapid and may spread throughout a continent, as occurred previously in Europe and the United States 1. Although several other carbapenemase-producing bacteria (KPC, GES, SPM, VIM) are reported to cause outbreaks or have endemic behavior in some Latin American institutions, the NDM enzyme has only been described in a few locations^2^. In Brazil, the enzyme has already been described in some states, such as Rio Grande do Sul, São Paulo, Paraná, Rio de Janeiro, Santa Catarina, Bahia, and Pernambuco^3^^-^ 9.

Immunosuppressed patients are particularly susceptible to infection by multidrug-resistant bacteria, including solid-organ transplant patients, perhaps because this population is frequently exposed to health facilities, invasive devices, and antimicrobial agents^10^^-^ 13. However, there are few published reports of NDM-producing bacterial infections in kidney transplant recipients^14^^-^ 16.

Hospital do Rim is a major kidney transplant center in Brazil and accepts patients from about 200 dialysis clinics in the country. The identification of carbapenem-resistant enterobacteria by the production of the enzyme KPC was first performed in our institution in 2009, but no cases of resistance by the production of the New Delhi Metallo-beta-lactamase (NDM) enzyme have been identified yet. Here, we report 4 cases of NDM-producing carbapenem-resistant enterobacterial strains identified in urine or rectal swab cultures from kidney transplant recipients at our center. We also conducted a cross-sectional surveillance 30 days after the hospital outbreak by rectal swab culture from all inpatients to assess the spread of this multidrug-resistant bacteria, totaling 101 examined patients. This project was approved by the local ethics committee (CAAE 15815419.7.0000.5505).

### Case Reports

Case 1 - A 68-year-old man who had been in hemodialysis for 3 years in a clinic located 20 km from the Hospital do Rim underwent a kidney transplant from a deceased donor. In the 8^th^ postoperative period, he presented dysuria, and urine culture was collected for investigation. Carbapenem-resistant *Klebsiella pneumoniae* (*_bla_* KPC-positive PCR) was isolated and the patient received meropenem associated with amikacin for 7 days, being discharged at the 20^th^ postoperative period. After 11 days, he developed graft dysfunction (creatinine of 1.89 to 2.61 mg/dL) with a new carbapenem-resistant *Klebsiella pneumoniae* isolation with *_bla_* KPC and *_bla_* NDM-positive PCR. He received endovenous amikacin and meropenem, but according to the sensitivity profile of the culture, meropenem was changed to oral phosphomycin (Table 1). The infection was completely resolved after 3 weeks of antibiotic treatment, and renal function returned to baseline. No urine culture was collected after treatment.

**Table 1:** Sensitivity profile of cultured isolated NDM-producing enterobactéria

Antibiotics	Case 1	Case 2	Case 3	Case 4
Imipenem	R	R	NT	R (>8)
Ertapenem	R	R	NT	R (>4)
Meropenem	R	R	NT	R (>8)
Ciprofloxacin	R	S	NT	I (2)
Norfloxacin	R	S	NT	NT
Sulfametoxazole- Trimethoprim	R	R	NT	S (<1/19)
Nitrofurantoin	R	I	NT	S (<16)
Amikacin	S	S	NT	I (32)
Gentamicin	S	S	NT	S (4)
Cefepime	R	R	NT	R (>16)
Ceftriaxone	R	R	NT	R (>32)
Colistin	S	S	NT	S (<1)
Phosphomycin	S (24 mcg/mL)	S (32 mcg/mL)	NT	S
Polymyxin	S (0.3 mcg/mL)	S (0.3 mcg/mL)	NT	S

S: Sensitive; R: Resistant; I: Intermediary; NT: Not tested.

Case 2 - A 60-year-old woman had been undergoing hemodialysis for three years in a city located 100 km from Hospital do Rim. Despite a history of recurrent urinary tract infections, she had not been hospitalized in the last 12 months and had never been evaluated for recurrent urinary tract infection before transplantation. She underwent a kidney transplant from a deceased donor, whose urine had no bacterial growth at the time of organ retrieval. The postoperative period was uneventful and she was discharged 9 days after transplantation with preserved graft function (serum creatinine of 1.10 mg/dL). Nine days after hospital discharge, she presented dysuria and mild renal dysfunction (creatinine of 1.46 mg/dL). Ciprofloxacin was started empirically. A few days later, urine culture showed growth of carbapenem-resistant *Klebsiella pneumoniae* with positive *_bla_* NDM (Table 1), evolving with symptoms and renal dysfunction resolution (creatinine 0.97 mg/dL). The medical team decided to maintain the antibiotic for 7 days. After 3 weeks, *Klebsiella pneumoniae* with positive *_bla_* NDM was isolated in a urine culture collected for microbiological control, but no therapy was initiated because the patient was asymptomatic. Subsequent urine cultures were negative. After transplantation, urinary tract infections no longer occurred.

Case 3 - A 29-year-old man who had been in hemodialysis for 3.5 years at a clinic 20 km from Hospital do Rim underwent a kidney transplantation from a living donor. Four days after surgery, the patient had an accidental contact with a patient colonized by carbapenem-resistant *Klebsiella pneumoniae*. A surveillance rectal swab was collected and resulted in the isolation of the bacteria with PCR positive for *_bla_* NDM. However, as the patient was asymptomatic, the medical team decided not to prescribe any treatment. Kidney function was stable, and the patient was discharged without complications.

Case 4 - A 38-year-old man, who underwent a kidney retransplant from a deceased donor seven years earlier was being treated for disseminated cryptococcosis with oral fluconazole when he was hospitalized for investigation of consumptive syndrome and fever. After six days of hospitalization, as the patient continued to have fever, a urine culture was collected from which *Enterobacter aerogenes* was isolated with positive PCR for *_bla_* NDM. Treatment with oral phosphomycin and intravenous meropenem for 14 days was prescribed. The symptoms improved and urine culture after treatment was negative. The patient remained hospitalized due to disseminated cryptococcosis and his clinical condition continued to worsen despite appropriate treatment for urinary tract infection. The patient required intensive care, but died 60 days after hospitalization due to the disseminated cryptococcosis.

None of the four cases described above were in the same inpatient unit during hospitalization.

### Cross-sectional surveillance of ndm-producing enterobacterial colonization

A cross-sectional analysis was performed in all inpatients 30 days after the identification of the last case to assess the NDM-producing enterobacterial dissemination in the hospital. We collected surveillance rectal swabs from 101 inpatients, and no new cases were detected. In addition, after 4 years of follow-up, no new diagnosis of infection with an NDM-1 producing multidrug-resistant strain was made in urine or rectal swab cultures collected at our center.

## Discussion

Preventing the spread of multidrug-resistant bacteria is an important issue worldwide. Detection of the NDM-1 enzyme-producing bacteria is a major new challenge for infectologists due to their great potential for spread and few therapeutic options. Due to its broad-spectrum drug resistance, NDM-1 strains are only susceptible to polymyxin, aminoglycosides, or the combination of two carbapenems with phosphomycin^8^^,^ 10 ^,^ 15.

The incidence of carbapenemase-producing enterobacteria infection in solid organ transplant recipients is 3 to 10%, with a mortality rate reaching 30%^13^. This incidence is of concern because kidney transplantation limits the use of nephrotoxic antimicrobials such as aminoglycosides reducing the available therapeutic options. There are no guidelines for the correct choice of antimicrobial agent and treatment duration, but there are reported cases of resolution of infection after treatment with phosphomycin alone or in combination with carbapenems lasting 14 days in these patients^14^^-^ 16. Although new antimicrobials such as ceftazidime-avibactam are good options for the therapy of infections caused by KPC-producing strains, they have no action against NDM-1 strains.

The bactericidal activity of phosphomycin involves the inhibition of proteoglycan synthesis of the bacterial cell wall, increasing cell membrane permeability of gram-positive and gram-negative bacteria^17^. Phosphomycin combined with other antimicrobials has shown good therapeutic results, sometimes better than its isolated use, as demonstrated by clinical and in vitro studies 2 ^,^ 18 ^,^ 19.

Of the 4 patients reported with NDM-1 positive culture, two of them developed an infection and were adequately treated with negative control cultures, and the other two patients were not treated because they were asymptomatic and also had subsequent negative urine cultures. No new colonization was identified in the cross-sectional screening in 101 inpatients, and no new cases of urinary NDM-1 infection were recorded at the hospital after a 4-year follow-up.

In this context, NDM-1 strains are particularly dangerous for several reasons: (a) most plasmids detected in these bacteria are transferable and capable of broad rearrangement, suggesting widespread horizontal transmission and flexibility among bacterial populations; (b) there is a lack of a standardized and widely accepted phenotypic test for routine detection of metallo-beta-lactamases (MBLs); (c) there is likely to be a high prevalence of undetected asymptomatic carriers, particularly in patients with chronic diseases who are frequently in the hospital setting; (d) there is a lack of effective, toxicity-favorable antibiotics for the treatment of multi-drug resistant NDM-1-expressing bacteria.

In addition, antibiotic resistance is spread by the misuse of antibiotics and by inadequate infection prevention and control. However, some measures could be taken to contain the spread of multidrug-resistance bacteria at hospital level, as recommended by the World Health Organization (WHO)^20^. Key steps include improving continuous surveillance for multidrug-resistance bacteria, promoting medical education on the rational use of antibiotics based on current guidelines, providing information on the impact of antibiotic resistance, promoting preventive measure such as handwashing, and cleaning of instruments and environment to the health care team and patients, and guiding patients to use antibiotics as directed.

## Conclusion

Surveillance and control of infections caused by multidrug-resistant strains in hospitals, especially those treating immunosuppressed patients, should be continuous and rigorous so that actions can be promptly taken to control multidrug-resistant bacteria outbreaks when they occur. The cases described here shed light to the high exposure to multidrug-resistant bacteria in hospitals specialized in solid organ transplantation, which receive recipients from different regions of the country, most of them on hemodialysis. In addition, these hospitals receive organs taken from deceased donors hospitalized in intensive care units, often for extended periods. Finally, according to the current knowledge, the use of phosphomycin alone or preferably in combination with non-nephrotoxic antimicrobial agents should be the therapy of choice for NDM-1 infections in solid organ recipients.
